# 879. Intravenous to Oral Antibiotic Switch in Patients Hospitalized with Community-Acquired Pneumonia

**DOI:** 10.1093/ofid/ofac492.072

**Published:** 2022-12-15

**Authors:** Abhishek Deshpande, Ning Guo, Peter B Imrey, Michael Rothberg

**Affiliations:** Cleveland Clinic, Cleveland, Ohio; Cleveland Clinic, Cleveland, Ohio; Cleveland Clinic, Cleveland, Ohio; Cleveland Clinic, Cleveland, Ohio

## Abstract

**Background:**

Community-acquired pneumonia (CAP) is a leading cause of hospital admissions and antimicrobial use worldwide. The ATS/IDSA guidelines recommend switching antimicrobials to per-oral once patients are clinically stable. Using a large national inpatient database, we assessed switch therapy practices across hospitals and their associations with outcomes in patients hospitalized with CAP.

**Methods:**

We included adult patients admitted with CAP and initially treated with intravenous (IV) antibiotics at 642 US hospitals from 2010–2015. Switching was defined as discontinuation of IV antibiotics and starting oral antibiotics without delay. Patients switched on or before hospital day 3 were considered early switchers. We conducted subgroup analysis restricted to patients initially treated with either 3^rd^ generation cephalosporin/macrolide or quinolone monotherapy, indicating non-severe inpatient CAP. In a propensity-adjusted analysis, we compared length-of-stay (LOS), in-hospital 14-day mortality, late deterioration (ICU transfer), hospital costs, total antibiotic days, and *C.difficile* infection between early switchers and others. We also compared adjusted outcomes across hospital switching rate quartiles.

**Results:**

Of 378,041 CAP patients, 116,118 (31%) were switched to oral antibiotics before discharge, and 21,784 (6%) were switched early. Hospital rates of early switching ranged from 3%-47%. Figure 1 summarizes the estimated effects of early switching among patients initially treated with cephalosporins/macrolide or quinolone monotherapy. Figure 2 shows the switch rate for patients within each hospital quartile, stratified by predicted mortality. Within each quartile, patients at higher predicted risk for mortality were less likely to be switched.

**Conclusion:**

In this large multihospital cohort, most patients were not switched to oral antibiotics before discharge and < 6% were switched early. Early switching was not associated with worse outcomes and was positively associated with shorter LOS and total days on antibiotics. Patients at hospitals in the top quartile of early switch rates did not suffer worse outcomes and had shorter LOS and total antibiotic duration. Our findings suggest clinicians could switch more patients early without compromising outcomes.

**Disclosures:**

**Abhishek Deshpande, MD, PhD**, Clorox: Grant/Research Support|Merck & Co., Inc., Kenilworth, NJ, USA: Advisor/Consultant|Merck & Co., Inc., Kenilworth, NJ, USA: Stocks/Bonds|Seres Therapeutics: Grant/Research Support.

**Figure 1:**
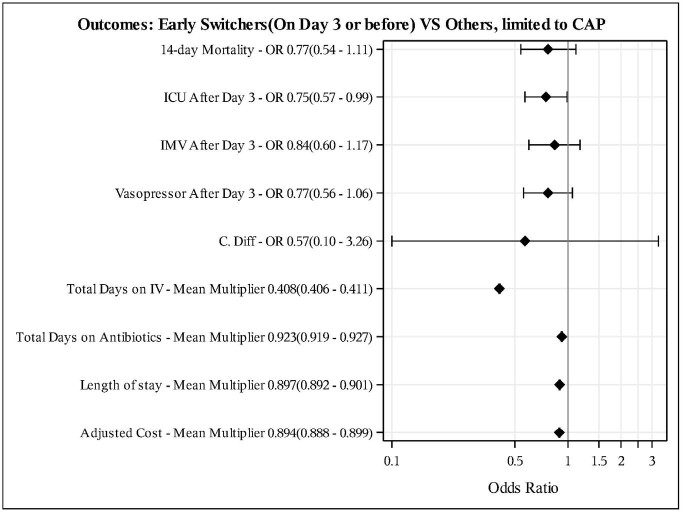

**Figure 2:**
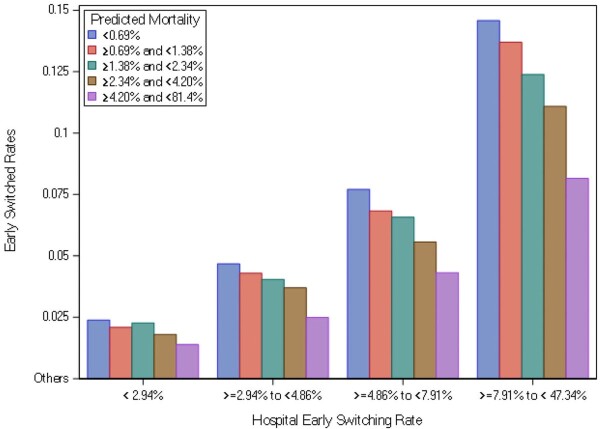
Bar chart of hospital rates of early switching (y axis) grouped by switching quartile and further stratified by quintiles of predicted mortality

